# Supplementary material for: The Association of Dietary Intake with Arterial Stiffness and Vascular Ageing in a Population with Intermediate Cardiovascular Risk—A MARK Study
Source: Nutrients. 2022 Jan 7;14(2):244. doi: 10.3390/nu14020244 (PMC8778402; doi:10.3390/nu14020244)
Supplement: Supplementary file 1 [file nutrients-14-00244-s001.zip › nutrients-1530444-supplementary.pdf]

Table S1. Lifestyles, cardiovascular risk factors and cardiovascular diseases in men according to degree of vascular aging

	Without EVA (1354)		With EVA (170)		p value
**Lifestyles**	**Mean or N**	**SD or (%)**	**Mean or N**	**SD or (%)**	
Years of smoker, (years)	31.83	12.64	32.98	13.99	0.552
Smoker. n (%)	440	32.5)	46	(27.1)	0.163
Alcohol. (gr/W)	100.91	129.81	110.73	154.02	0.276
Risk consumption (n. %)	252	(18.6)	32	(18.8)	917
Total FA. (METs/m/W)	2864.19	2765.17	2861.22	3195.96	0.990
Sedentary. n (%)	1119	(82.6)	150	(88.2)	0.080
MD. (total score)	5.13	1.76	4.84	1.90	0.045
Adherence MD. n (%)	705	(52.1)	76	(44.7)	0.042
DQI. (total score)	38.71	3.12	37.80	3.26	<0.001
Adherence DQI. n (%)	732	(54.1)	71	(41.8)	0.003
**Conventional risk factors**					
Age. (years)	61.01	8.15	61.91	7.77	0.164
SBP. (mmHg)	137.40	15.97	152.50	19.36	<0.001
DBP. (mmHg)	85.07	10.10	90.43	11.82	<0.001
BP. (mmHg)	52.32	13.60	62.07	15.76	<0.001
MBP. (mmHg)	102.27	10.58	110.86	12.74	<0.001
Hypertension. n (%)	1015	(75.0)	157	(92.4)	<0.001
Antihypertensive drugs. n (%)	671	(49.6)	97	(57.1)	0.029
Total cholesterol. (mg/dl)	220.74	39.01	217.67	38.20	0.333
LDL cholesterol. (mg/dl)	139.20	34.33	131.42	33.00	0.006
HDL cholesterol. (mg/dl)	47.78	11.57	48.74	14.72	0.321
Triglycerides. (mg/dl)	148.58	97.95	170.51	155.15	0.012
No-HDL cholesterol. (mg/dl)	173.00	38.36	168.93	38.12	0.192
Atherogenic index (mg/dl)	4.84	1.30	4.71	1.25	0.223
Dyslipidemia. n (%)	867	(64.0)	102	(60.0)	0.311
Lipid–lowering drugs. n (%)	372	(27.5)	47	(27.6)	0.962
FPG. (mg/dl)	106.55	32.66	117.38	41.38	<0.001
HbA1c. (%)	6.01	1.09	6.39	1.30	<0.001
Diabetes mellitus. n (%)	418	(30.9)	75	(44.1)	<0.001
Hypoglycaemic drugs. n (%)	234	(17.3)	55	-324	<0.001
Height. cm	164.64	9.240	163.86	9.545	0.060
Weight. kg	169.80	6.70	168.76	7.36	0.226
WC. (cm)	102.87	10.64	103.53	9.55	0.439
BMI. (kg/m^2)	29.07	3.98	28.96	3.76	0.743
Obesity. n (%)	450	(33.2)	60	(35.3)	0.605
Abdominal obesity. n (%)	700	(52.0)	97	(57.1)	0.222
Plasma creatine. (mg/dl)	0.93	0.18	0.99	0.48	0.002
GFR (mL/min/1.73 m^2)	87.02	14.41	84.94	18.00	0.119
CVR. SCORE scale. (%)	3.41	2.59	4.66	3.48391	<0.001
**Cardiovascular diseases**					
Renal disease. n (%)	0	(0.0)	2	(1.2)	<0.001
Peripheral arteriopathy	0	(0.0)	11	(6.5)	<0.001
Heart failure	0	(0.0)	11	(6.5)	<0.001
**Vascular function**					
baPWV. (m/s)	14.27	1.89	19.26	3.57	<0.001

Values are means ± standard deviations for continuous data and number and proportions for categorical data.
Risk alcohol consumption in women were ≥140 g/week and in men ≥210 g/week. Sedentary if the moderate physical activity performed is <675 METs minute / week or the intense physical activity <420 METs minute / week. Definition American Heart Association. 2007. Adherence MD ≥ 5. Adherence DQI ≥ 39.

N. number; SD. standard deviation; gr/W. grams/week; FA. physical activity; METs/m/W. basal metabolic rate/minute/week; MD. mediterranean diet; DQI. diet quality index; SBP. systolic blood pressure; DBP. diastolic blood pressure; BP. pulse pressure; MBP. mean blood pressure; LDL. low–density lipoprotein; HDL. high–density lipoprotein; FPG. fasting plasma glucosa; HbA1c. glycosylated hemoglobin; WC. waist circumference; BMI. body mass index; CVR. cardiovascular risk; GFR. glomerular filtration; baPWV. Brachial-Ankle pulse wave velocity.
p value: differences between men and women.

Table S2. Lifestyles, cardiovascular risk factors and cardiovascular diseases in women according to degree of vascular aging

	Without EVA (856)		With EVA (95)		p value
**Lifestyles**	**Mean or N**	**SD or (%)**	**Mean or N**	**SD or (%)**	
Years of smoker. (years)	23.03	43.14	28.56	134.38	0.385
Smoker. n (%)	212	(24.8)	12	(12.6)	0.007
Alcohol. (gr/W)	30.90	12.87	28.05	13.85	0.339
Risk consumption (n. %)	44	(5.1)	6	-63	0.626
Total FA. (METs/m/W)	1824.28	1651.19	1754.08	1958.60	0.700
Sedentary. n (%)	774	(90.4)	84	(88.4)	584
MD. (total score)	5.32	1.65	5.25	1.49	0.670
Adherence MD. n (%)	467	(54.6)	47	(49.5)	0.386
DQI. (total score)	38.91	2.92	38.67	3.05	0.461
Adherence DQI. n (%)	501	(58.6)	54	(56.8)	0.743
**Conventional risk factors**					
Age. (years)	61.61	7.03	62.53	6.70	0.226
SBP. (mmHg)	132.68	16.03	149.06	22.53	<0.001
DBP. (mmHg)	82.32	9.40	87.57	10.46	<0.001
BP. (mmHg)	50.36	12.95	61.53	18.75	<0.001
MBP. (mmHg)	98.85	10.35	107.82	12.82	<0.001
Hypertension. n (%)	539	(63.0)	84	(88.4)	<0.001
Antihypertensive drugs. n (%)	442	(51.6)	62	(65.3)	0.013
Total cholesterol. (mg/dl)	234.85	42.66	224.05	45.49	0.020
LDL cholesterol. (mg/dl)	143.26	35.70	133.98	37.38	0.017
HDL cholesterol. (mg/dl)	53.01	14.28	51.91	11.78	0.472
Triglycerides. (mg/dl)	137.88	79.71	145.01	68.20	0.402
No-HDL cholesterol. (mg/dl)	181.82	43.58	172.14	45.45	0.041
Atherogenic index (mg/dl)	4.69	1.34	4.50	1.29	0.197
Dyslipidemia. n (%)	629	(73.5)	66	(69.5)	0.396
Lipid–lowering drugs. n (%)	263	(30.7)	35	(36.8)	0.244
FPG. (mg/dl)	106.21	34.73	127.56	43.82	<0.001
HbA1c. (%)	6.13	1.21	6.93	1.43	<0.001
Diabetes mellitus. n (%)	292	(34.1)	57	(60.0)	<0.001
Hypoglycaemic drugs. n (%)	179	(20.9)	43	(54.3)	<0.001
Height. cm	156.48	6.37	155.09	6.10	0.054
Weight. kg	72.46	13.64	71.43	14.00	0.486
WC. (cm)	97.72	12.77	98.15	12.04	0.754
BMI. (kg/m^2)	29.59	5.29	29.66	5.28	0.898
Obesity. n (%)	350	(40.9)	37	(38.9)	0.742
Abdominal obesity. n (%)	671	(79.0)	78	(82.1)	0.593
Plasma creatine. (mg/dl)	0.71	0.13	0.71	0.16	0.917
GFR (mL/min/1.73 m^2)	88.60	12.86	88.24	14.47	0.800
CVR. SCORE scale. (%)	1.98	1.33	2.68	1.79	<0.001
**Cardiovascular diseases**					
Renal disease. n (%)	0	(0.0)	0	(0.0)	
Peripheral arteriopathy	0	(0.0)	2	(0.2)	0.010
Heart failure	0	(0.0)	1	(0.1)	0.100

**Vascular function**					
baPWV. (m/s)	14.40	1.99	19.81	2.41	<0.001

Values are means ± standard deviations for continuous data and number and proportions for categorical data.
Risk alcohol consumption in women were ≥140 g/week and in men ≥210 g/week. Sedentary if the moderate physical activity performed is <675 METs minute / week or the intense physical activity <420 METs minute / week. Definition American Heart Association. 2007. Adherence MD ≥ 5. Adherence DQI ≥ 39.
N. number; SD. standard deviation; gr/W. grams/week; FA. physical activity; METs/m/W. basal metabolic rate/minute/week; MD. mediterranean diet; DQI. diet quality index; SBP. systolic blood pressure; DBP. diastolic blood pressure; BP. pulse pressure; MBP. mean blood pressure; LDL. low–density lipoprotein; HDL. high–density lipoprotein; FPG. fasting plasma glucosa; HbA1c. glycosylated hemoglobin; WC. waist circumference; BMI. body mass index; CVR. cardiovascular risk; GFR. glomerular filtration; baPWV. Brachial-Ankle pulse wave velocity.
p value: differences between men and women.

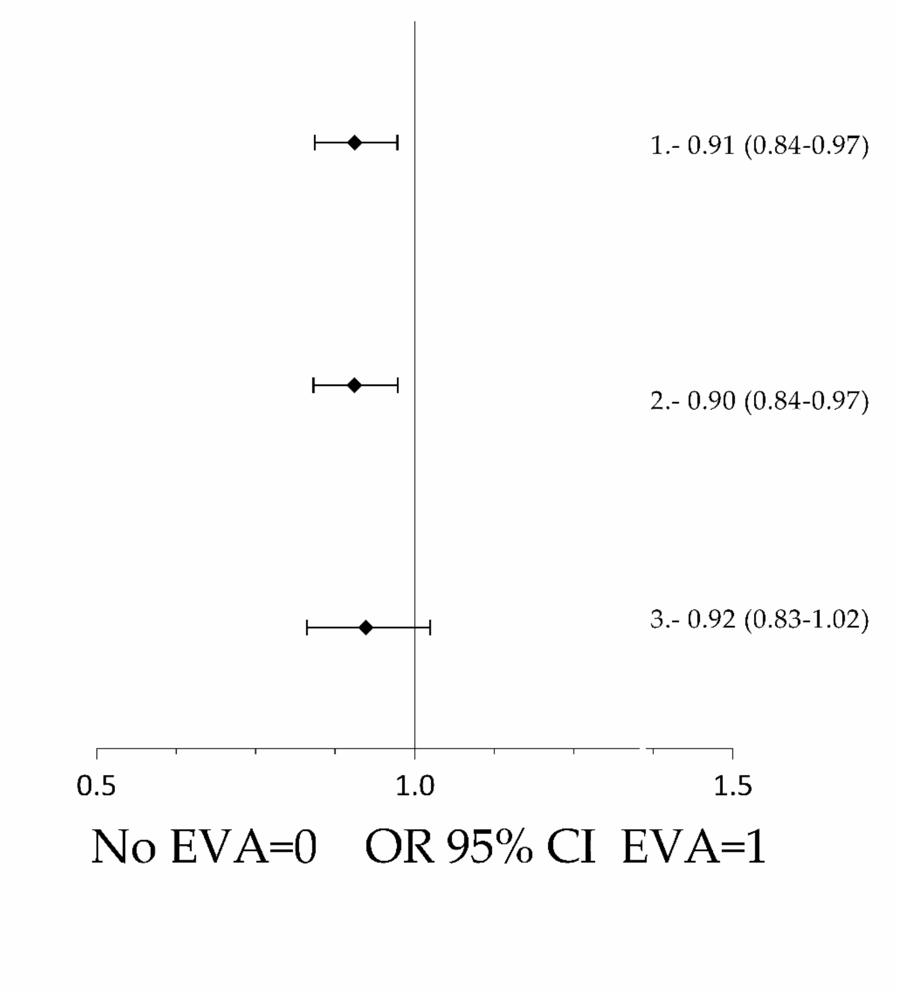

Figure S1. Bars show OR (odds ratio) and 95% CI. Association between arterial ageing and DQI score by food categories. Dependent variable: the presence of early vascular ageing (EVA=1) versus non-EVA (non-EVA=0). Independent variables: adherence to food categories of DQI (1 = Yes, 0 = No). Adjustment variables: age, sex (0 = woman; 1 = man), being a smoker, being sedentary, being a risk drinker, and the intake of antihypertensive, lipid-lowering or glucose-lowering drugs (no risk factor or no intake of drugs=0, having the risk factor or intake of drugs=1). DQI, diet quality index; EVA, early vascular ageing. The first category includes 8 food groups (bread, vegetables, fruits, milk and yougurt, rice and pasta, vegetable oils, alcoholic drinks, and cereals). The second category includes seven food groups (meat, sausages, cheese, sweets, animal fat, other vegetable oils and fast food). The third category includes three food groups (fish, legumes and walnuts).

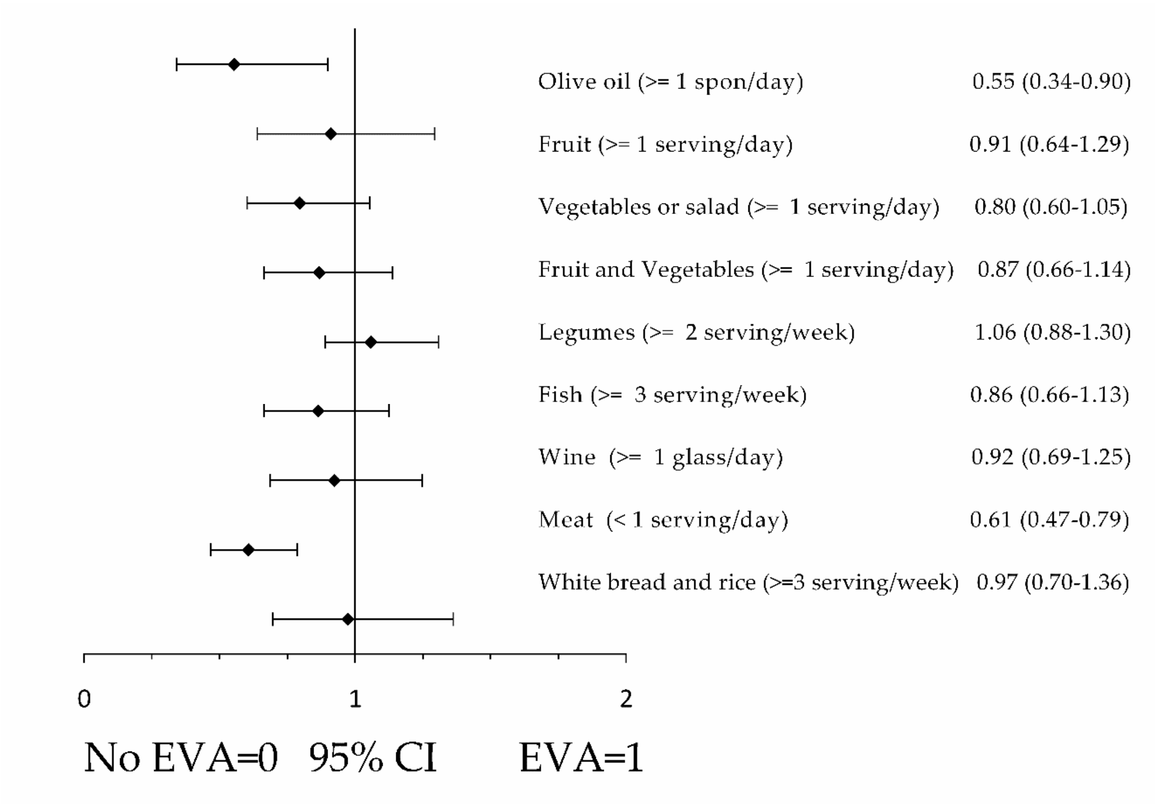

Figure S2. Bars show OR (odds ratio) and 95% CI. Association between arterial ageing and adherence to the components of the MD. Dependent variable: the presence of early vascular ageing (EVA=1) versus non-EVA (non-EVA=0). Independent variables: adherence to the 9 components of the MD (1 = Yes, 0 = No). Adjustment variables: age, sex (0 = woman; 1 = man), being a smoker, being sedentary, being a risk drinker, and the intake of antihypertensive, lipid-lowering or glucose-lowering drugs (no risk factor or no intake of drugs=0, having the risk factor or intake of drugs=1). DQI, diet quality index; EVA, early vascular ageing.
